# Association of HLA-DQB1∗05:02 and DRB1∗16 Alleles with Late-Onset, Nonthymomatous, AChR-Ab-Positive Myasthenia Gravis

**DOI:** 10.1155/2012/541760

**Published:** 2012-10-09

**Authors:** Manuela Testi, Chiara Terracciano, Annalisa Guagnano, Giuseppe Testa, Girolama A. Marfia, Eugenio Pompeo, Marco Andreani, Roberto Massa

**Affiliations:** ^1^Laboratory of Immunogenetics, IME Foundation at Tor Vergata Polyclinic Hospital, 00133 Rome, Italy; ^2^Department of Neurosciences, Tor Vergata University of Rome, Via Montpellier 1, 00133 Rome, Italy; ^3^Department of Thoracic Surgery, Tor Vergata University of Rome, Via Montpellier 1, 00133 Rome, Italy; ^4^Department of Neurosciences, IRCCS Fondazione S. Lucia, 00179 Rome, Italy

## Abstract

An association of several HLA alleles with myasthenia gravis (MG) has been reported. Aim of this work was to analyze the HLA allele profile in a survey of 76 unselected Italian MG patients and in a subgroup characterized by disease onset after the age of 50 years, absence of thymoma, and presence of antiacetylcholine receptor antibodies. We defined this subgroup by the acronym LOAb. Typing was performed at low resolution for HLA-A, -B, and -DRB1 loci with sequence-specific oligonucleotide probe (PCR-SSO); at high resolution for HLA-DQB1 locus by PCR with sequence-specific primers (PCR-SSPS). HLA allele frequencies were compared with 100 healthy controls. No correlation was observed between MG and the studied HLA class I alleles. On the contrary, a strong positive association was found for the HLA class II alleles DQB1∗05:02 (*P*_*c*_ = 0.00768) and DRB1∗16 (*P*_*c*_ = 0.0211) in the LOAb subgroup (*n* = 27) of MG patients. Association between DQB1∗05:02 and some subtypes of MG has been previously reported but not in patients with the LOAb characteristics. Therefore, the HLA allele DQB1∗05:02 might be considered as a susceptibility marker for LOAb among Italians.

## 1. Introduction

Myasthenia gravis (MG) is a heterogeneous autoimmune disorder affecting neuromuscular transmission. Subtypes of the disease differ in age at onset, symptom distribution, autoantibody profiles, and associated thymic abnormality. Appropriate recognition of the different clinical subtypes may help to determine management strategies and prognosis. MG is usually considered to be more prevalent among young women; however, recent epidemiological studies have shown an increased incidence of late-onset (LO) MG, the factors responsible for this change being still unknown [1–3].

Family studies have reported an increased risk of MG in relatives of the patients, suggesting involvement of genetic factors in the development of the disease. Different studies have investigated the association of HLA genes with MG, reporting nonhomogenous results in terms of association in different populations and disease subgroups [4–9]. No clear association with specific HLA alleles has been reported so far in LOMG. We analyzed the HLA allele profile in a series of Italian MG patients and in a particular subgroup, characterized by the absence of thymoma, the presence of antiacetylcholine receptor antibodies (AChR-Ab) and onset of the disease after the age of 50. In accordance with recent studies, patients sharing these features represent the most numerous subgroup among our population [1, 2].

## 2. Patients and Methods

### 2.1. Patients

Seventy-six consecutive, unrelated, Italian MG patients, recruited by the Department of Neurosciences, Tor Vergata University of Rome, entered in the survey. All patients gave informed consent to inclusion in the study, which was approved by the local Ethics Committee. The diagnosis of MG was made on the typical history and signs of fluctuating weakness of voluntary muscles, a decremental pattern on repetitive nerve stimulation, and/or an increase in jitter on single-muscle fiber studies [10]. Age at disease onset, presence of serum anti-AChR or anti-muscle-specific kinase (MuSK) antibodies, and presence of thymoma, as assessed by chest CT scan, were additional criteria used for identifying MG subtypes. Considering the bimodal age pattern, the separation between early onset (EO) and LO in nonthymomatous MG patients was placed at the age of 50 [2]. Surgical thymectomy was performed on 35 patients. On histopathological examination, 12 had a thymoma, 11 had thymic hyperplasia, and 12 had thymic atrophy. During completion of this study, two patients deceased, one of a metastatic lung cancer, and the other of a myocardial infarct.

### 2.2. HLA Typing

DNA was obtained from blood samples by a fully automated system (Maxwell, Promega, Milano, Italy). Low-resolution typing for HLA-A, -B, and -DRB1 loci was performed by using polymerase chain reaction-sequence-specific oligonucleotide (PCR-SSO) (Luminex, One Lambda, Canoga Park, CA, USA); high resolution typing for DQB1 was performed by using polymerase chain reaction-sequence-specific primers (PCR-SSPS) (Olerup, Stockholm, Sweden; Invitrogen, Carlsbad, CA, USA). 

### 2.3. Statistical Analysis

HLA allele frequencies were estimated by direct counting in patients and in 100 healthy controls of Italian origin. To compare the differences between the allele frequencies in the control and MG groups, a 2 × 2 contingency table analysis was performed using the Fisher exact test. The strength of association between HLA alleles and MG was estimated by odds ratio and 95% confidence intervals. *P *< 0.05 was considered to be statistically significant. *P* values were corrected (*P*_*c*_) for multiple comparisons according to the Bonferroni method. 

## 3. Results and Discussion

Demographic and clinical data of our patient population are summarized in Table 1. Briefly, a moderate majority of females (58%) over males was observed. This difference was due to a large preponderance of females with early-onset disease, whereas a late onset was more common among males. Twelve (16%) patients had a thymoma. Other categories of MG patients were represented by those with anti-MuSK-Ab (9%), by early-onset patients with (EOAb, 17%) or without (EO, 13%) AChR-Ab, and late-onset patients with (LOAb, 36%) or without (LO, 9%) AChR-Ab. 

We did not find any association between MG and HLA class I alleles, neither in the entire survey or in the LOAb subgroup of 27 patients. On the contrary, a strong positive association was found for the HLA class II alleles DQB1*05:02, (*P*_*c*_  = 0.00768) and DRB1*16 (*P*_*c*_  = 0.0211) among the 27 patients belonging to the LOAb subgroup (Table 2). 

In this study, a positive association between the DQB1*05:02 and DRB1*16 alleles with MG was found in a subset of patients, stratified for age at onset higher than 50 years, absence of thymoma, and presence of AChR-Ab. Several associations have been found between HLA antigens or alleles and MG in various ethnic groups, in which the highest risk for MG was consistently conferred to the class II HLA loci. In particular, the DQB1*05:02 has been found to be associated with MG in various populations such as different types of Italian MG patients (except for those with LO) [5] or male Turkish patients [7] or with anti-MuSK-positive MG [9]. Moreover, DQ5, the serologic equivalent of DQB1*05:02, has been shown to be strongly associated with anti-MuSK MG in a Dutch cohort [8]. Our findings revealed associations between DQB1*05:02 and DRB1*16 with a previously unreported subgroup of patients, representing the most common MG subtype diagnosed in the last two decades [1–3]. 

Although this hypothesis needs to be confirmed, the lack of association with MG in the whole survey may be due to the presence of patient subgroups not sharing the same HLA alleles, such as the AChR-Ab-negative or the thymomatous patients. Therefore, the apparent discrepancy between our and other results may be ascribed to the variability of different MG subsets and to the changing pattern of incidence of MG [2]. Interestingly, the occurrence of MG with different antibody specificity (AChR and MuSK) has been described in a mother and daughter sharing the DQ5 antigen [11]. 

We might assume that the DRB1*16-DQB1*05:02 haplotype is at increased risk for MG, since the linkage disequilibrium between these alleles is known; it is, however, possible that only DQB1*05:02 is a susceptibility marker for some forms of MG, considering the higher statistical significance of its association. On the other hand, it must be considered that the HLA genes involved might merely be markers of nearby non-HLA genes responsible for the susceptibility or resistance effects. Further studies in this direction need to be carried out in order to elucidate the full spectrum of immunogenetics of myasthenia gravis.

## Figures and Tables

**Table 1:** Demographic and clinical features of the MG patient population.

Disease subtypes	Patients	Males	Females
T-MG	12 (16%)	3	9
MuSK-MG	7 (9%)	2	5
EO-MG	10 (13%)	2	8
EOAb-MG	13 (17%)	1	12
LO-MG	7 (9%)	5	2
LOAb-MG	27 (36%)	19	8

Total	76	32 (42%)	44 (58%)

T-MG: thymomatous myasthenia gravis; MuSK-MG: anti-MuSK-positive myasthenia gravis; EO-MG: early-onset myasthenia gravis; EOAb-MG: early-onset myasthenia graviswith anti-AChR antibodies; LO-MG: late-onset myasthenia gravis;LOAb-MG: late-onset myasthenia graviswith anti-AChR antibodies.

**Table 2:** HLA class II allele frequency analysis in the LOAb-MG patients subgroup.

Alleles	Healthy controls		Patients			Statistical analysis		
	*n*	Allelic frequencies	*n*	Allelic frequencies	OR	95% CI	*P*	*P*_*c*_
HLA-DRB1								
*01	15	0.075	6	0.111			NS	
*015	16	0.080	4	0.074			NS	
*016	7	0.035	9	0.167	5.5143	1.0407–15.596	**0.0016**	**0.0211**
*03	22	0.110	1	0.019			NS	
*04	18	0.090	6	0.111			NS	
*011	56	0.280	9	0.167			NS	
*012	3	0.015	0	0.000			NS	
*013	21	0.105	1	0.019			NS	
*014	9	0.045	7	0.130			NS	
*07	21	0.105	10	0.185			NS	
*08	8	0.040	0	0.000			NS	
*09	1	0.005	0	0.000			NS	
*10	3	0.015	1	0.019			NS	

Total alleles	200	1	54	1				

HLA-DQB1								
*02	39	0.195	9	0.167			NS	
*03	85	0.425	17	0.315			NS	
*04	8	0.040	0	0.000			NS	
*05:01	18	0.090	6	0.111			NS	
*05:02	8	0.040	10	0.185	5.4545	2.0354–14.617	**0.0009**	**0.00768**
*05:03	9	0.045	7	0.130			NS	
*05:04	0	0.000	0	0.000			NS	
*06	33	0.165	5	0.093			NS	

Total alleles	200	1	54	1				
